# A Study of the Impact of Thirteen Celebrity Suicides on Subsequent Suicide Rates in South Korea from 2005 to 2009

**DOI:** 10.1371/journal.pone.0053870

**Published:** 2013-01-16

**Authors:** King-wa Fu, C. H. Chan

**Affiliations:** 1 Journalism and Media Studies Centre, The University of Hong Kong, Hong Kong, China; 2 Department of Social Work and Social Administration, The University of Hong Kong, Hong Kong, China; University of Western Brittany, France

## Abstract

A number of ecological studies have found a pattern of increasing suicide rates after suicides of several Asian entertainment celebrities. However, the finding may be subject to positive outcome bias where cases with no perceived impact may be routinely excluded. In this study, we deploy interrupted time-series analysis using ARIMA transfer function models to investigate systematically the impact of thirteen celebrity suicides on subsequent suicide rates in South Korea. We find that three out of eleven cases were found to be followed by a significant increase in suicide rate, while controlling for seasonality, secular trends, and unemployment rates. Such significant increases could last for nine weeks. Non-significance cases may be attributable to the small amount of media coverage, the “displacement” effect of preceding case, and the negative connotation of celebrity deaths. We therefore conclude that whether or not the impacts were detected may be largely conditioned by various contextual factors. Current evidence based on ecological studies is insufficient to draw a firm conclusion. Further studies using multiple approaches should be developed.

## Introduction

Research in past decades has demonstrated that celebrity suicide and its associated media reporting can stimulate a subsequent rise in the suicide rate [1], [2], [3]. Such rise is found to be more salient among specific gender, age group, or among those who died by the same suicide method as the celebrity did [4], [5], [6]. A systematic review of ten international studies supports a significant increase of the suicide rate in a month after incidents of celebrity suicides, and indicating no direct evidence of publication bias [7]. Computer simulation results suggest that mass suicide cluster via mass media transmission may be due to copying celebrities’ suicidal behaviour [8]. Recent studies have extended the investigation into the influence of the suicides of Asian entertainment celebrities and have reconfirmed the impact [4], [5], [6]. However, all these studies examined single or self-selected cases of celebrity suicide. The selection criteria are often unreported. Researchers may selectively report cases that have higher perceived impact, whereas other cases with low perceived impact may be consistently excluded. This may give rise to publication bias and at worst may mislead the conclusion. Such type of positive outcome bias has been found in observational study design [9]. Therefore in this study, we propose to deploy a systematic approach to selection of cases and test the effect hypothesis in a unified statistical model.

When a number of celebrity suicide cases are systematically selected and jointly investigated, this approach may induce a carryover effect [10] or displacement effect [11] – whether or not the impact of an incident of celebrity suicide may heighten or lessen the subsequent effect of the following incidents. Moreover, a few studies have suggested a non-immediate impact of celebrity suicide that lasts for more than six months [5], [12]. In this study, the displacement effect and the longer-term effect are hypothesized to be particularly profound when a chain of celebrity suicides is clustered in a short time period. For example, seven Korean entertainment celebrities killed themselves in seven months from September 2008 to March 2009 [13]. The suicide rate in South Korea, which was reported to climb up to 31.0 per 100,000 in 2009, was ranked as one of the highest globally [14].

With this background, this study aims to systematically investigate the impact of entertainment celebrity suicides on the overall suicide rate in South Korea and secondly to test the displacement effect and longer-term effect hypotheses.

## Methods

### Ethics Statement

Because of the fact that no personal data were involved, this study was exempted from ethical review by the Human Research Ethics Committee for Non-Clinical Faculties, The University of Hong Kong.

### Data Collection

We followed the definition from *Webster’s Dictionary*; celebrity was defined as a person who is “widely known.” Media archives were therefore used as a mean to identify the suicides of widely known entertainers. A systematic search for media reports of the suicides of the South Korean entertainment celebrities between January 1, 2003, and December 31, 2009, was conducted. The search covered major media outlets in South Korea including television channels KBS and MBC and the newspapers Chosun Ilbo, Joongang Ilbo, and Donga Ilbo. We deployed same searching strategy as previous study [5]: news stories were retrieved by Korean keyword searches for “suicide death”, “actor’s suicide”, “actress’ suicide”, “singer’s suicide” or “star’s suicide” for the study period. The search was conducted by a native Korean undergraduate student by checking news on the media’s official websites where provide keyword search in the media archives. The search results were later reviewed by the two authors. Based on the search, thirteen cases of celebrity suicides were identified in the study period. Details of the cases of celebrity suicide are summarized in Table 1. In the study, incidents of celebrity suicide were mapped based on the week of the first report of the suicide. As there were three reports of celebrity suicide within a single week, these three cases were aggregated and treated as a single incident (Incident 5), resulting in a total of 11 incidents of celebrity suicides. In addition, to estimate the media coverage of the celebrity suicides, counts of the media stories containing the name of celebrity in a week before and after his or her death were summarized in Table 1.

**Table 1:** Summary of the details of the suicides of the South Korean Entertainment Celebrities between 2005 and 2009.

Incident no.	Surname ofcelebrity	Date of death	Gender	Age	Method ofsuicide	CorrespondingICD-10 code	Description	News count (7 days before the incident)	News count (7 days after the incident)	Media reports on the causes of death
1	Lee	Feb 22 2005	F	24	Hanging	X70	Actress	5	718	depression
2	Nee	Jan 21 2007	F	26	Hanging	X70	Singer	25	894	depression and work pressure
3	Jong	Feb 10 2007	F	26	Hanging	X70	Actress	0	1411	depression
4	Ahn	Sept 8 2008	M	36	Charcoal	X67	Actor	0	2290	financial problem
5	Choi	Oct 2 2008	F	39	Hanging	X70	TV and movie actor	35	2252	distressed by online rumor that linked her to Ahn's death.
5	Jang	Oct 3 2008	F	26	Hanging	X70	Transgender actress	0	59	distressed by breakup with her boyfriend
5	Kim	Oct 6 2008	M	23	Hanging	X70	Gay fashion model	0	56	distressed by the consequence of his "come-out"
6	Lee	Dec 1 2008	M	29	Hanging	X70	Band singer	0	61	investment loss
7	Kim	Jan 17 2009	M	30	Hanging	X70	Actor	0	45	depression
8	Jang	Mar 7 2009	F	29	Hanging	X70	TV actress	2	2526	depression and involved in sex scandal in the Korean entertainment
9	Lee	Mar 12 2009	M	38	Hanging	X70	Singer	0	65	financial problem
10	Woo	Apr 27 2009	F	24	Hanging	X70	Model and actress	0	139	depression
11	Kim	Nov 19 2009	F	20	Hanging	X70	Model	0	401	depression

* Incident 5 represents 3 cases of entertainment celebrity as they all occurred in the same calendar week.

Suicide mortality data for South Korea between January 1, 2003, and December 31, 2009, was obtained from the National Statistical Office of Korea [15]. Death counts assigned the codes from X64 to X80 in the International Classification of Diseases, Tenth Revision, (ICD-10) were included in the study. For each death, information was available on date of death, age, gender, and suicide method as coded employing ICD-10 [16]. The data were amalgamated into weekly suicide counts. In addition, the monthly unemployment rate of South Korea between December 2002 and November 2009 was also collected from the National Statistical Office of Korea [15].

### Analytic Plan

To examine the effect of celebrity suicides on weekly suicide counts, interrupted time series analysis was performed. Instead of ordinary regression analysis, the Box-Jenkins autoregressive integrated moving average (ARIMA) model [17] was used because it takes into account the autocorrelation of the weekly data.

The eleven incidents of South Korean celebrity suicide (Table 1) were considered to be predictors in the statistical models and the outcome variable was the time-series data of weekly suicide counts in South Korea. Use of actual suicide counts instead of suicide rates for the analysis was based on consideration of the low population growth within the study period. The population growth rate between 2003 and 2009 in South Korea ranged from 0.21% to 0.5%. In addition, the monthly unemployment rate of South Korea was included in the analysis to take into account its impact on suicide deaths [18].

The analysis was organized in the following steps. First, we examined whether an incident of celebrity suicide can have an immediate impact on suicide rate. Specifically, the eleven incidents of celebrity suicide were simultaneously entered into the ARIMA model to estimate the immediate impact on suicide counts. The equation representing the model is.
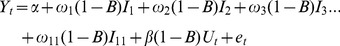
(1)Where *Y_t_* is the number of suicide deaths at week *t*, *α* denotes the average number of suicide deaths per week, *e_t_* denotes the random error, and *(1 − B)* is the backshift operator, *U_t_* denotes the unemployment rate at week *t*, and *I_1,_ I_2,_ I_3_* to *I_11_* represent the eleven incidents of celebrity suicide at their “immediate impacted week”, which is defined as follows: Incidents 1,2,4,5,6,10 that occurred on Day 1 (Sunday) to Day 3 (Tuesday) of a week, their “immediate impacted week” were defined as the same week of the incident; Incidents 3,7,8,9,11 that occurred on Day 5 (Thursday) to Day 7 (Saturday) of a week, their “immediate impacted week” was chosen as the week followed. The *β* is the coefficient estimating the effect of unemployment rate on suicide counts, and ω_1_ to ω_11_ are the corresponding coefficients measuring the immediate impact of the incident of celebrity suicide.

To explore the carryover effect of the incidents, decline functions were incorporated into the model. A smaller value of the decline function implies that the rate of decline of the impact is slower, suggesting a longer-lasting impact. The function that measured the immediate impact as well as the carryover or displacement effect is expressed as
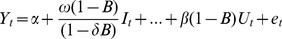
(2)


In equation 2, *ω* and *δ* are the corresponding coefficients measuring the immediate impact and the decline rate respectively.

To evaluate the longer-term impact of celebrity suicide on suicides, dummy step functions (i.e. value of 0 before the incident and a value of 1 from the incident to end of the study period) were inputted. This modification in the model is shown in the following equation.
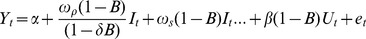
(3)


In equation 3, *ω_p_* and δdenote the immediate impact of the incident and its rate of decline, and *ω_s_* is the coefficient representing the longer-term impact of the incident.

Box et al.’s standard procedure was followed for model fitting [17]. In the diagnostic check, plots of autocorrelation function (ACF) and partial autocorrelation function (PACF) were utilized to determine stationary characteristic of the time-series model. The Ljung-Box chi-square test was also used to test the absence of autocorrelation in the residual. All of the models were built using the maximum likelihood method.

The weekly suicide counts for the entire South Korean population were first fitted with the aforementioned models for hypothesis testing. Previous studies have found that changes in suicide rates are age-, gender-, and suicide method-specific [5]; therefore, sub-group analyses were also undertaken for detecting whether there were similar patterns. Specifically, suicide data were stratified into various time-series based on the demographic characteristics of the population, including genders, age groups (<20 years of age, 20 to <40 years of age, 40 to <60 years of age, 60 or above), and suicide methods (killing themselves using the same method as the celebrity and killing themselves by other methods). Three second order subgroups (age-gender, gender-method, and age-method specific) and one third order subgroup (age-gender-method specific) analyses were also examined.

All analyses were calculated using the *R* statistical package version 2.14 [19]. For the analysis of the time-series of suicide counts for the entire population, the statistical test of significance was set at the 5% level. For the sub-group analysis, in order to avoid inflation of the Type I error due to multiple comparisons, the statistical test was revised to a more conservative level. Utilizing the Bonferroni approach, the significance level was adjusted according to the total number of independent tests involved. As 12 time-series data were tested in the subgroup analyses, the significance level for the subgroup analysis was adjusted to the level of 0.4% (Bonferroni correction for the significance level: 0.05/12 = 0.00412).

## Results

**Figure 1:**
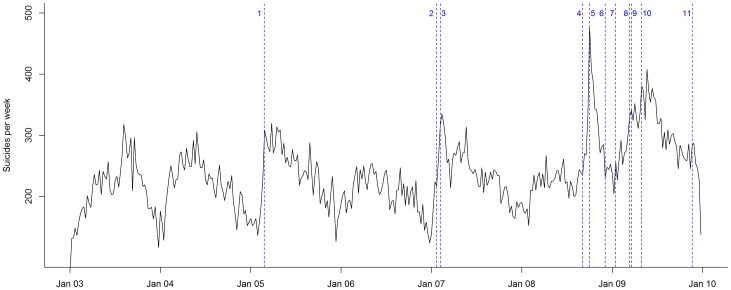
Overall suicide counts in South Korea between 1 January 2003 and 31 December 2009. Remarks: The vertical dashed lines marks the week in which a celebrity died.

Time-series of weekly suicide deaths for the South Korea population between January 1, 2003, and December 31, 2009, are plotted in Figure 1. Within the study period, there were 85,751 suicide deaths. Of those deaths, 56,770 (66%) were males and 28,981 (34%) were females. Suicide by hanging, strangulation, or suffocation (ICD-10 X70) was the most common suicide method in South Korea, accounting for 45% (n = 38,771) of the total suicide cases. The totals for suicide counts in those aged less than 20, 20 to 39, 40 to 59, and 60 years or above groups were 6,501 (7%), 19,670 (23%), 30,053 (35%), and 29,913 (35%), respectively.

### Impacts of Celebrity Suicides

Table 2 summarizes the immediate impact of the eleven incidents of celebrity suicide. Three incidents (incident 1, 3, and 5) were found to have a significant impact on the overall suicide count, after adjusting for the secular trend, seasonality, and the unemployment rate. Concerning the suicide death of EJ Lee (incident 1), its immediate impact was observed to be significant in the main model (ω = 65.2, 95%CI = 22.9, 107.5, p = 0.003). In particular, an increase of 65 suicide deaths at the week of incident 1 should be attributed to the suicide death of EJ Lee. The decline rate estimate showed a significant increase of the suicide deaths remained for 3 weeks. However, the estimate of the decline rate was not statistically significant (decline rate = 0.2, p = 0.149). When using step function to model longer-term impact, significant increase of suicide cases was detected (ω = 85.1, 95%CI = 39.8, 130.3, p<0.004), suggesting that the number of suicides increased rather than decreased after the week of incident 1.

**Table 2:** The immediate impact of the celebrity suicide.

	Incident 1(F,24,H)^c^	Incident 2(F,26,H)^c^	Incident 3(F,26,H)^c^	Incident 4(M,36,C)^c^	Incident 5(F,39,H;F,26,H,M,23,H)^c^	Incident 6(M,29,H)^c^	Incident 7(M,30,H)^c^	Incident 8(F,29,H)^c^	Incident 9(M,38,H)^c^	Incident 10(F,24,H)^c^	Incident 11(F,20,H)^c^
***Main model***											
	65.2 (22.9, 107.5)^**^	22.3 (−18.2, 62.8)	50.7 (5.4, 96.1)*	−23.2 (−63.6, 17.3)	149.3 (105.5, 193.1)^**^	− 36.3 (−76.7, 4.1)	− 22.8 (−63.5, 17.9)	15.9 (−27.0, 58.8)	−11.8 (−54.2, 30.6)	37.9 (−3.2, 79.0)	29.1 (−11.6, 69.8)
***1^st^ order subgroup***											
Aged less than 20	6.3 (1.2, 11.4)	1.0 (−4.1, 6.2)	6.0 (0.9, 11.1)	−0.1 (−5.3, 5.0)	3.6 (−1.6, 8.7)	−1.3 (−6.5, 3.9)	−2.2 (−7.4, 3.0)	2.8 (−2.5, 8.0)	3.8 (−1.5, 9.0)	6.1 (0.9, 11.4)	7.7 (2.5, 13.0)
Aged 20 to 39	39.9 (20.9, 59.0)^**^	−3.1 (−21.2, 15.1)	22.9 (3.2, 42.7)	18.0 (−0.1, 36.1)	60.5 (41.1, 79.5)^**^	−15.9 (−33.9, 2.1)	−11.5 (−29.6, 6.5)	−4.8 (−23.8, 14.1)	−7.7 (−26.6, 11.3)	−1.0 (−19.1, 11.2)	9.5 (−8.6, 27.6)
Aged 40 to 59	34.0 (13.0, 55.0)	24.6 (4.6, 45.7)	17.8 (−3.7, 39.3)	−19.2 (−39.9, 1.6)	73.9 (52.5, 95.2)^**^	−13.1 (−33.8, 7.7)	0.3 (−20.5, 21.2)	22.4 (1.2, 43.5)	10.2 (−10.9, 31.4)	2.3 (−18.4, 23.1)	−4.9 (−25.8, 16.0)
Aged 60 or above	−9.4 (−34.0, 15.2)	8.8 (−15.7, 33.4)	7.1 (−17.4, 31.6)	−24.2 (−48.4, 0.1)	20.3 (−4.4, 45.1)	1.5 (−23.0, 26.1)	−10.5 (−34.6, 13.5)	4.0 (−20.2, 28.1)	−11.1 (−35.3, 13.0)	23.0 (−1.0, 46.9)	16.9 (−8.1, 41.8)
Male	25.9 (−6.5, 58.3)	10.9 (−21.4, 42.3)	19.2 (−13.3, 51.7)	−18.2 (−50.4, 14.0)	35.4 (2.8, 68.0)	−29.6 (−61.8, 2.7)	−18.8 (−51.2, 13.7)	8.2 (−25.4, 41.9)	7.2 (−26.1, 40.6)	34.3 (1.4, 67.2)	15.5 (−16.9, 48.0)
Female	32.2 (11.2, 53.2)	12.9 (−7.9, 33.7)	25.7 (4.2, 47.3)	−4.7 (−25.5, 16.1)	106.1 (84.1, 128.0)^**^	−4.6 (−25.5, 16.3)	−2.2 (−23.1, 18.7)	10.0 (−12.2, 32.2)	−11.2 (−33.3, 10.8)	5.7 (−15.2, 26.6)	16.7 (−4.2, 37.5)
Same method	50.6 (20.6, 80.7)	22.4 (−5.9, 50.6)	26.6 (−1.0, 54.1)	– a	120.8 (90.1, 151.4)^**^	−27.5 (−55.6, 0.5)	−15.0 (−43.2, 13.2)	1.3 (−28.8, 31.4)	−4.3 (−34.3, 25.8)	−4.2 (−32.5, 24.0)	5.9 (−22.5, 34.2)
Other methods	10.0 (−19.4, 39.5)	11.8 (−17.7, 41.3)	7.4 (−21.8, 36.6)	−6.4 (−36.6, 23.8)	10.9 (−19.2, 41.0)	−5.3 (−35.4, 24.7)	−9.4 (−40.0, 21.1)	16.5 (−15.5, 48.5)	−4.3 (−36.1, 27.5)	46.2^**^ (15.6, 76.9)	24.0 (−6.7, 54.7)
***2^nd^ order subgroup***											
Same gender and age group	25.3 (14.1, 36.5)^**^	7.4 (−3.9, 18.7)	19.4 (6.7, 32.1)^**^	10.1 (−2.8, 23.0)	56.9 (45.1, 68.7)^**b^	−13.7 (−26.7, −0.8)	−7.6 (−20.5, 5.3)	4.5 (−7.5, 16.6)	−0.9 (−14.0, 12.2)	−4.9 (−16.2, 6.4)	11.6 (0.3, 23.0)
Same gender and method	37.0 (22.2, 51.2)^**^	5.0 (-9.3, 19.4)	20.9 (7.0, 34.9)^**^	– a	84.5 (69.2, 99.9)^**^ b	−19.5 (−40.0, 0.9)	−12.1 (−32.7, 8.5)	13.5 (−1.6, 28.6)	−1.4 (−22.4, 19.5)	5.0 (−9.8, 19.8)	5.6 (−8.7, 19.9)
Same method and age	30.9 (15.8, 46.0)^ **^	9.5 (−5.2, 24.2)	9.6 (−5.6, 24.9)	– a	53.8 (38.0, 69.7)^ **^	−14.0 (−28.4, 0.3)	−4.3 (−18.9, 10.2)	−5.0 (−20.3, 10.3)	−8.9 (−24.2, 6.4)	−0.8 (−15.3, 13.6)	1.9 (−12.7, 16.4)
***3^rd^ order subgroup***											
Same age and gender and method	22.3 (13.5, 31.0)^ **^	6.7 (−2.1, 15.5)	10.7 (3.2, 18.1)^ **^	– a	45.8 (36.1, 55.4)^**^ b	−10.7 (20.5, −0.9)	−2.6 (−12.4, 7.2)	8.8 (−0.5, 18.0)	−3.6 (−13.8, 6.6)	3.7 (−5.1, 12.5)	7.1 (−1.6, 15.8)

* p<0.05, **p<0.004.

a we could not identify an adequate model to produce the estimate.

b Estimates of the female population are reported.

c Inside the bracket (Gender, Age, Suicide Method), Gender: M-Male, F-Female; Age – in years; Suicide Method: H- Hanging, C – Charcoal burning.

The first order sub-group analysis showed that the impact of incident 1 was more profound among population with similar age ranges (aged 20-to-39 and aged 40-to-59), same sex (female) as Lee, and same method of suicide (ICD-70) as she did. However, when we adopted the adjusted significance level, i.e. 0.4%, for multiple group comparisons, the impact of incident 1 was significant only among those aged 20 to 39 (p<0.004). Higher orders of the sub-group analysis confirmed significant rises in suicides in populations with matched characteristics (same age-gender, gender-method, age-method, or age-gender-method). When examining longer-term impact for the sub-group analysis, significant increase of suicides were found among those who aged 20 to 39 (ω = 46.9, 95%CI = 27.3, 66.4, p<0.004), females (ω = 41.1, 95%CI = 18.6, 63.6, p<0.004), and those who died by hanging (ω = 75.5, 95%CI = 43.0, 108.0, p<0.004). Estimates of the step function for incident 1 were also found to be significant in all higher-order sub-group analyses.

The suicide of Jong (incident 3: a 26 year-old female actress died by hanging) was also followed by a significant increase of suicide deaths in the overall population. Its impact remained significant only for a week (decline rate = 0.28, *p*<0.05). Significant increases of suicide deaths were detected in some second order sub-groups: same gender and age group (p<0.004) and same gender and method (p<0.004). However, estimates of the decline rate in the sub-group analyses were found to be non-significant.

Compared to Lee and Jong’s deaths, the suicides of Choi, Jang, and JH Kim (incident 5) was found to have the strongest immediate impact on the suicide deaths. An increase of 149 suicides was estimated to be attributable to the incident 5 and the duration of the impact remained significant for 9 weeks (the impact at the 10th week: *ω* = 44.5, 95%CI = −2.8, 84.7). The sub-group analyses revealed that the significant impact of these three celebrity suicides was evident among those who aged 20-to-39 (*p*<0.004), aged 40-to-59 (*p*<0.004), female (*p*<0.004), and dying by hanging (*p*<0.004). The impact on subgroups was estimated to last for 6 (aged 20–39: decline rate = 0.43, p<0.004) to 12 weeks (female: decline rate = 0.32, p<0.004). Findings of the higher-order sub-group analyses were similar to the first order sub-group analysis and the duration of the impact ranged from 10 (same gender and age group: decline rate = 0.39, p<0.004) to 14 weeks (same gender and method: decline rate = 0.34, p<0.004).

To model the longer-term impact of the celebrity deaths, we explored the impact of incident 5 with another model using both the step-function and the decline function. Based on the new model, we found that the immediate impact of the incident 5 explained 65% of the overall impact of the incident 5 (ω = 117.2, 95%CI = 56.8, 177.7, *p*<0.004). The immediate impact was estimated to last for 4 weeks (decline rate = 0.38, p<0.004). The remaining 35% of the overall impact of the incident 5 were estimated to be marginally significant in a longer-term basis (step function: ω = 61.9, 95%CI = −0.9, 124.8, *p* = 0.054). Similar results were observed in the first order sub-group analyses. For instance, significant immediate impacts were detected among those aged 20-to-39 (ω = 43.9, p<0.004), female (ω = 88.7, p<0.004), and dying by hanging (ω = 82.9, p<0.004). Moreover, a significant longer-term rise in suicide deaths was discovered among those who died by hanging (ω = 88.8, p<0.004).

## Discussion

This study investigated the impacts of thirteen suicides of South Korean entertainment celebrities on the country’s suicide rate within a seven-year study period. Our systematic case selection approach differentiates from the one usually used in previous studies that celebrity cases were mostly single events and self-selected. Using the robust time series ARIMA model that has been commonly deployed for analyzing public health data [20], we estimated the effects of celebrity suicide while adjusting for the impacts of seasonality and secular trends on the suicide rate and controlling for unemployment rate, which is often treated as a potential confounder in previous studies [4], [5]. Consistent with previous studies [4], [5], [6], subgroup analyses further indicate that the rises in suicides were markedly observed among those who were the same age or gender as the celebrity and who used the same suicide method, whereas no such increases were found among groups of the older ages, opposite gender and among those who used other suicide methods.

Based on the time-series data of the overall suicide counts, we found that only three incidents of celebrity suicide (incident 1, 3 and 5) were followed by significant increases in suicide rate. Most incidents (incident 2, 4 and 6–11) did not lead to any subsequent rise. One plausible explanation may be due to relatively smaller coverage of some incidents in the media (incident 6, 7, 9, 10, and 11). Previous studies show the scale of impact is linked to the amount of media coverage and the ways of presentation [21], [22]. Another plausible explanation may be the consequence of the profound impacts of incident 1, 3 and 5. Previous studies have suggested the “displacement hypothesis,” positing that a strong effect may cause suicides to occur earlier than would have been the case otherwise and consequently the subsequent impacts are saturated or diminished [11], [23]. Specifically, the impact of incident 5 (suicides of three celebrities in the same week) was very salient and had caused a shape increase in suicide rate in the subsequent period. Although there were additional reports of celebrity suicides of incident 6 in the subsequent period, its main effect estimate was negative and did not lead to a statistically significant increase. Nevertheless, a significant drop in suicides among same age-gender-method group was observed. “Displacement hypothesis” may be also attributable to the negative and insignificant estimate for the group aged 20 to 39 (Incident 2). Another possible reason would be that some celebrity deaths were given a negative connotation by the public. For example, JY Jang’s death (incident 8) was reportedly linked to a sex scandal in the Korean entertainment industry. Despite a huge amount of media interest, as identification is a prerequisite for celebrity influence [3], the negative connotation may discount the impact and partly explain why there was no marked rise in suicide rate after her death.

With this result, we argue that the impact of celebrity suicides on an individual’s tendency to kill oneself is indeed a complex psychosocial process rather than a universal stimulus-response effect. Informed by social psychology and media research [24], the impact is likely to be conditioned by situational factors and cognitive characteristics, i.e. amount of media coverage, whether or not there is preceding impact, and the personal characteristics and history of the celebrity, and is subject to individual difference and various audience’s orientations. Unfortunately, most of the previous ecological studies and quantitative approach do not seem to be able to completely disentangle the complex nature of the impact of celebrity suicide on suicide rate. Understanding of such a conditional mechanism remains a research gap. To further advance the body of knowledge in this area, future research should explore an underlying mechanism of the effect and should not solely count on using an ecological design or a quantitative approach. Theoretically speaking, an information processing model should be developed to examine an individual’s reaction to media accounts of celebrity suicide, for example, exploring a model of the media’s influence on an individual’s suicidal ideation [25].

Methodologically speaking, a qualitative approach, such as interviewing suicide survivors after a celebrity suicide, may provide information-rich verbatim material to help unfold unexplored contextual factors [26], [27], with a goal of testing observations drawn from quantitative studies.

As the study simultaneously examined the impact of the eleven incidents of celebrity suicide, this approach allowed us to directly compare the magnitude, the decline rate and the duration of the impacts. Results showed that in the incident where there were multiple celebrity suicides (incident 5), its impact on the suicide rate was stronger and lasted longer than other single-case incidents (incident 1), indicating a possible dose-response relationship. This observation is consistently found in the analyses of overall suicide deaths as well as the subgroup analysis. Previous studies have documented the dose-response relationship between imitational suicides and newspaper distribution [21].

While majority of previous studies support that celebrity suicide exerts only a short-lived negative impact on the suicide rate with an effect typically lasting no longer than four weeks, this study adds to previous evidence [5], [12] supporting that the impact of celebrity suicide on the suicide rate may have lasted longer than that. We found that weeks after incident 1 and incident 5, increase in overall suicide deaths was continuously observed. Nonetheless, this study showed that, although a large proportion of the impact of celebrity suicide would diminish over course, a small but significant proportion of the impact would remain and contribute to a subsequent rise in the suicide rate. This observation is parallel with a previous finding that the media may “sow the seeds of suicide in the distant future” [28]. This implies that celebrity suicide can impose non-immediate rise in overall suicide risk in the population level and public health measures should be therefore developed to mitigate the elevated risk. In individual level, a study has shown that celebrity suicide can have a long-term negative impact on individual’s suicidal ideation, especially those who have greater anxiety symptoms, fewer reasons for living, and more focus on irrational values. [12]. This further suggests that the impact of celebrity suicide would trigger individual’s suicidal thought over the course of a person’s life particularly when one is under adverse situation or in unhealthy mental condition. But this proposition must be subject to further confirmation in future study. Moreover, since this study is an observational study, other external factors, such as political, social, or economic changes in South Korea, may possibly account for the increase in suicide rate during the study period. One possible factor is the global financial crisis since 2008 [29].

Charcoal burning was an uncommon suicide method in South Korea before the occurrence of Incident 4. Previous study in Taiwan finds an increase in charcoal burning suicides after extensive media reporting on a singer’s suicide using the same method [30]. However, the estimates for “Same Method” related subgroup analysis (Incident 4) can not be computed because the number of charcoal burning suicides before the incident was too small for the ARIMA model to converge.

### Limitations

As in many previous studies using ecological design, we cannot ascertain whether individuals who died by suicide during the study period had actual exposure to the news of celebrity suicide. Nor we can ascertain that exposure to the news may directly contribute to their suicidal behaviour. However, in a previous study that investigated the impact of a South Korean celebrity’s suicide (incident 2 in this study) on suicide attempters [31], 89.2% of the participants reported an exposure to the media report of the suicide of that celebrity, and a quarter of them stated an influence of the news on their subsequent suicide attempts. In light of the observation, it seems legitimate for us to assume that a large proportion of the suicides in this study could have been due to exposure to media reports of celebrity suicide.

### Conclusion

We deployed a systematic approach to investigating the impact of a group of celebrity suicides in South Korea, totalling 13 over a five-year time period. We further analyzed the data rigorously in a time-series statistical model, suggesting that three incidents were followed by subsequent increases in suicide deaths. Such increases were found mostly among the same age or gender groups and the same suicide method group. This basically reconfirms established evidence of the impact of celebrity suicide. However, in such a systematic approach, we find that whether or not an impact is detected would have been conditioned by some contextual factors, e.g. the extent of media coverage or negative public connotation. Interaction between cases, that is to say displacement effect, may explain whether or not an effect was found. The effect of celebrity suicide on the suicide rate may have longer-term impact.

The major implication of this study is that, we argue, scholars in this research area should deploy more diverse approaches, both theoretically and methodologically, to examine a broader scope of contextual factors. Otherwise, the established ecological evidence may be challenged by other scholars, especially social scientists, who seek to explain phenomena by referring to social contexts and routinely reject the “media effect” assertion that is widely regarded as too mechanical and absolute. We call for more innovative research using a mixed-method approach to advance this field of study.
